# Pyelonephritis‐associated *Staphylococcus saprophyticus* bacteremia in an immunocompetent host: Case report and review of the literature

**DOI:** 10.1002/ccr3.5183

**Published:** 2021-12-05

**Authors:** Ahmad Matarneh, Gawahir A. Ali, Wael Goravey

**Affiliations:** ^1^ Department of Internal Medicine Hamad Medical Corporation Doha Qatar; ^2^ Department of Infectious Diseases Communicable Diseases Centre Hamad Medical Corporation Doha Qatar

**Keywords:** bacteremia, kidney calculi, pyelonephritis, *Staphylococcus saprophyticus*

## Abstract

*Staphylococcus saprophyticus* is one of the coagulase‐negative staphylococcus species. It is the second most frequent causative microorganism in acute uncomplicated urinary tract infections in young women. However, it is potentially capable of causing more invasive infections including bacteremia, particularly secondary to pyelonephritis. We present a young, previously healthy lady who presented with urinary symptoms and hemodynamic instability and was found to have multiple renal and ureteric calculi with pyelonephritis. Later, blood and urine cultures isolated methicillin‐resistant *S. saprophyticus*. The patient was successfully treated with a course of antibiotics targeting the organism with a favorable outcome. The clinical presentations and management of this rare entity of *S. saprophyticus* bacteremia‐related pyelonephritis are outlined. In addition, the literature on similar cases was reviewed to raise awareness and avoid devastating consequences.

## Graphical abstract

Bacteremia due to *Staphylococcus saprophyticus* is a rare occurrence and appears to represent the end of the spectrum of urinary tract infection with this organism. The timely identification of methicillin‐resistant *S. saprophyticus* and the use of targeted antimicrobials therapy is crucial to avoid consequences and treatment failure.

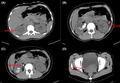

## 1 INTRODUCTION

*Staphylococcus saprophyticus* is a gram‐positive, novobiocin‐resistant, coagulase‐negative staphylococcus species.^1^ It is the second most frequent causative microorganism in acute uncomplicated urinary tract infections in young, sexually active women.^2^ However, it can present with a variety of complicated genitourinary tract infections which include prostatitis, pyelonephritis, and epididymitis.^3^ *Staphylococcus saprophyticus* bacteremia rarely complicates the involvement of the urinary tract, particularly in immunocompetent hosts.^4^ The clinical presentation and diagnosis are usually undistinguished *S. saprophyticus* bacteremia secondary to pyelonephritis from typical uropathogens.^5^

Typically, *S. saprophyticus* is sensitive to most antimicrobials used to treat UTIs. However, there is rising resistance of *S. saprophyticus* to empirically and commonly used antibiotics to treat cystitis, hence, rendering the management more challenging.^6^ Herein, we report an unusual highly resistant case of *S. saprophyticus* pyelonephritis leading to bacteremia in an otherwise healthy young female patient who was successfully treated with a course of vancomycin and daptomycin. In addition, we reviewed the literature for similar cases.

## 2 CASE PRESENTATION

A 28‐year‐old lady, previously well presented to the hospital with a 2 days history of fever, dysuria, and left flank pain. She had no chronic medical condition or previous similar episodes. On examination, vital signs showed a fever of 38.2 with normal BP 115/71 and HR of 87. Abdominal examination revealed suprapubic and costovertebral tenderness. Basic investigation revealed high inflammatory markers with pyuria and acute kidney injury as depicted in the below (Table 1).

**TABLE 1:** Basic laboratories

Detail	Value w/Units	Normal range
WBC	30 × 10^3^/μl	4.0–10.0
Hgb	15.4 gm/dl	13.0–17.0
Platelet	400 × 10^3^/μl	150–400
Absolute Neutrophil count Auto# (ANC)	28 × 10^3^/μl	2.0–7.0
Neutrophil Auto %	93.4%	
INR	1.2	
Urea	10.1 mmol/L	2.8–8.1
Creatinine	130 μmol/L	62–106
Bicarbonate	26 mmol/L	22–29
CRP	65	0–5

**FIGURE 1:**
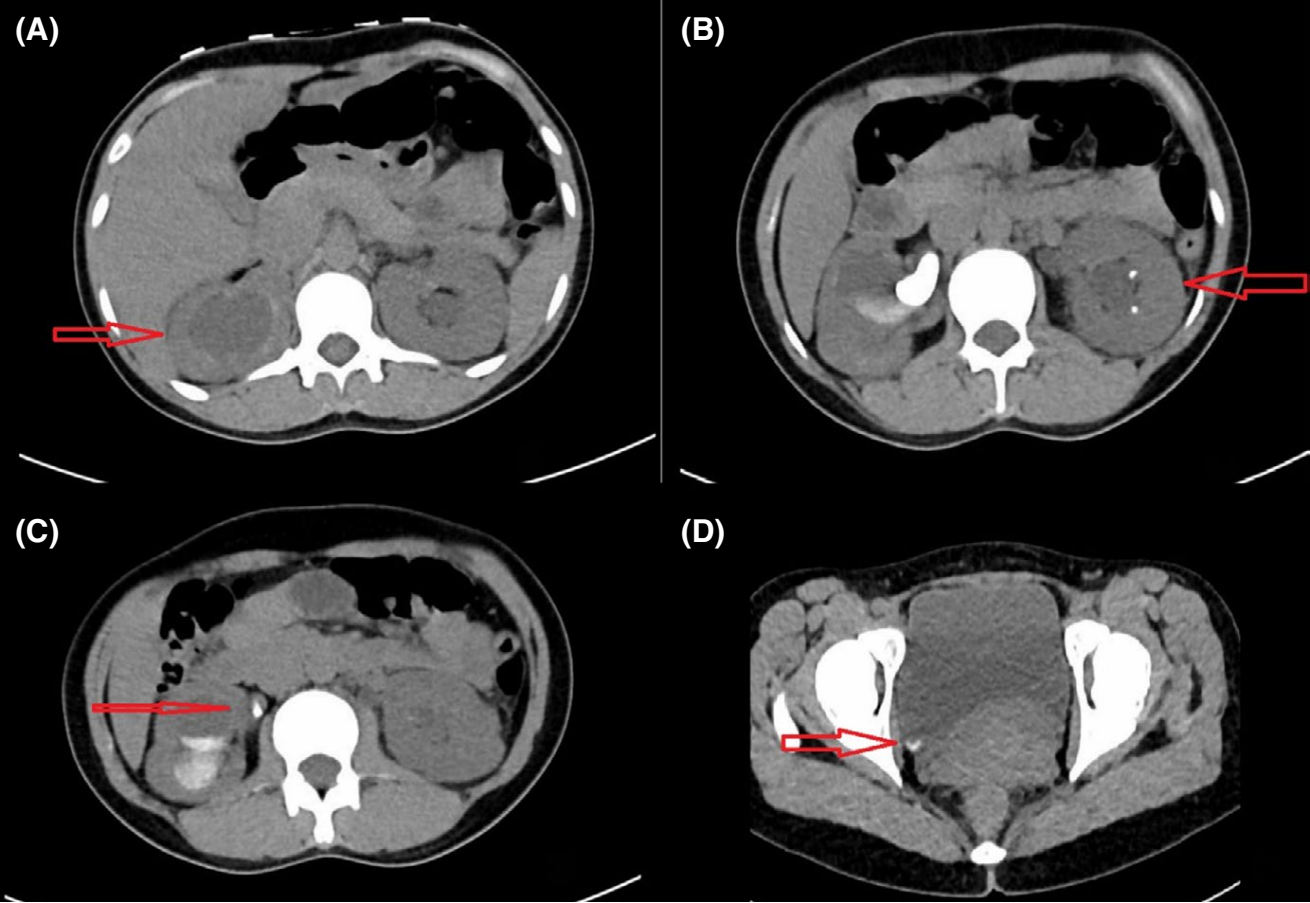
CT KUB findings of (A) Hydronephrosis, (B) Renal calculi with evidence of hydronephrosis, (C) Ureteric calculi, and (D) Urinary bladder stone

Computed tomography scan revealed bilateral renal calyceal and ureteric stones causing hydronephrosis and obstructive uropathy (Figure 1). The patient was started on meropenem as per local antibiogram for presumptive urosepsis awaiting the results of the cultures. Both blood and urine cultures grew highly resistant *staphylococcus saprophyticus* that was sensitive only to vancomycin, daptomycin, gentamycin, and linezolid. Hence, the patient's antibiotic was changed to vancomycin. An echocardiogram was done, and it ruled out cardiac vegetations, and repeated blood cultures after 3 days were negative. A bilateral percutaneous nephrostomy was inserted which relieved her urinary obstruction. Subsequently, the patient improved clinically with normalization of her kidney parameters. Following removal of the percutaneous nephrostomy after passing the stones, she was discharged on daptomycin for ease of outpatient administration to complete a total of 14 days of antibiotics. On follow‐up visits, she had no recurrence of her urinary symptoms, and normal kidney function was restored.

## 3 DISCUSSION

*Staphylococcus saprophyticus* is a gram‐positive organism that possesses multiple virulence factors, most importantly hemagglutinin and catalase. It usually attaches to the epithelium of the urogenital tract utilizing hemagglutinin and adhesins that help to anchor the bacteria to the cell wall allowing it to escape the immune system. Furthermore, catalase protects *S. saprophyticus* from being killed by reactive oxygen species.^7^ The existence of a renal tract obstruction would favorably enable *S. saprophyticus* to ascend more proximally toward the renal pelvis causing pyelonephritis as in our case.^8^ *Staphylococcus saprophyticus* is a well‐recognized cause of uncomplicated cystitis after *E. coli* and has been isolated in 42% of young females with uncomplicated cystitis.^2^, ^9^ The usual course of uncomplicated *Staphylococcus saprophyticus* infection is mild and responds well to antibiotics either orally or intravenously in more severe cases.^3^ Nevertheless, complicated genitourinary infections including prostatitis, pyelonephritis, and epididymitis have rarely been reported in certain high‐risk patients.^8^ Many risk factors have been reported for complicated *Staphylococcus saprophyticus* UTIs such as immunocompromised hosts, obstructive nephrolithiasis, indwelling urethral catheters, and the presence of renal tract anomalies.^10^ However, it rarely causes infection in immunocompetent adults.

Although *Staphylococcus saprophyticus* is a well‐established cause of uncomplicated UTIs, it is pathogenic significance, role in complicated UTIs, and clinical significance when isolated from blood culture has not been well defined.^4^, ^10^ It has been postulated that *S. sapro* is of low virulence due to multiple reasons, including the absence of coagulase, unlike other staphylococci. Coagulase degrades fibrin and results in clotting activation, and coats the organism helping to escape phagocytosis. Furthermore, *Staphylococcus saprophyticus* lacks ATPase; hence, it is difficult to grow in low potassium environments such as the blood, whereas urine is a suitable medium because it is potassium contents.^8^ For these reasons, the pathogenicity of *S. saprophyticus* might be lower in the blood than in urine because of its physiological function and activity. Even though, when bacteremia occurs, the significance is still not well established, and further studies are required to delineate the course.^4^

*Staphylococcus saprophyticus* is generally sensitive to most antibiotics including beta‐lactams. However, some strains isolated from complicated UTIs were generally more resistant to broad‐spectrum antibiotics than those isolated from uncomplicated infections.^11^ This explains the high resistance profile isolated from our patient.

The optimal treatment for bacteremia due to *S. saprophyticus* is not yet well defined given it is a rarity. In our case, we treated the patient with antibiotics for 2 weeks given the highly resistant nature of the organism and the presence of urinary obstruction.^4^

Our search of the literature yielded a total of nine cases of *S. saprophyticus* bacteremia originating from the urinary tract (Table 2). Cases ranged between 14 and 53 years of age and were predominantly female. Half of them had underline urolithiasis while no immunosuppressed status was reported. Pyelonephritis is mostly involved in bacteremia rather than uncomplicated UTIs. Of the cases identified, only two cases reported some form of resistant *staphylococcus saprophyticus* as in our case. The duration of therapy ranged from 7 days to 21 days. All cases achieved complete recovery and clearance of the bacteremia with no complications (Table 2).

**TABLE 2:** Summary of previously reported cases of *S. saprophyticus* bacteremia originating from the urinary tract

	Case	Sex/age	Comorbidities	Predisposing factor	Source	Empirical treatment	Definite treatment	Duration of Abx	Outcome
1	Golledge^12^	F/14	None	Sexual activity	Pyelonephritis	Amoxicillin, cloxacillin,	penicillin	10 days	Recovered
2	Golledge^12^	F/49	None	Sexual activity/Uretric obstruction recurrent UTI	Pyelonephritis	Cephalothin, gentamicin	penicillin	7 days	Recovered
3	Glimaker^13^	F/19	None	Sexual activity	Pyelonephritis	Co‐trimoxazole	cloxacillin, flucloxacillin	3 weeks	Recovered
4	Glimaker^13^	F/33	None	Ureteric Stones Sexual activity	Pyelonephritis		Co‐trimoxazole	14 days	Recovered
5	Olafsen^14^	F/27	None	Pregnancy, ureteric calculus	Pyelonephritis	Ampicillin	amoxicillin	12 days	Recovered
6	Chen^15^	F/38	None	Unknown	Pyelonephritis		Gentamicin	14 days	Recovered
7	Lee^16^	F/38	None	Pregnancy	Urinary tract infection	cefazolin	cephalexin	7 days	Recovered
8	M Hofmans^17^	F/53	None	Ureterolithiasis	Urinary tract infection	Temocillin	ciprofloxacin	10 days	Recovered
9	Our case		None	Ureterolithiasis	Pyelonephritis	Meropenem	Vancomycin then Daptomycin	14 days	Recovered

## 4 CONCLUSION

Pyelonephritis‐associated *staphylococcus saprophyticus* bacteremia in an immunocompetent host is a rare clinical entity that demonstrates the ability of the organism to manifest as invasive infections. *S. saprophyticus* is generally sensitive to most antibiotics commonly used to treat uncomplicated community‐acquired UTIs; however, resistance strain is raising which necessitating caution when dealing with the invasive infection of this organism. The treatment should be guided by the pattern of antimicrobials sensitivity, and the optimal treatment duration remains unknown.

## CONFLICT OF INTEREST

The authors have no conflict of interest to declare.

## AUTHOR CONTRIBUTIONS

Ahmad Matarneh, Gawahir A. Ali, and Wael Goravey: Clinical care, literature review, and manuscript write up.

## ETHICAL APPROVAL

The case report was approved by Hamad medical corporation, MRC number MRC‐04‐21‐242.

## CONSENT

Written informed consent was obtained from the patient to publish this report in accordance with the journal's patient consent policy.

## Data Availability

The data that support the findings of this study are available from the corresponding author upon reasonable request.
